# Design and Processing of a Novel Chaos-Based Stepped Frequency Synthesized Wideband Radar Signal

**DOI:** 10.3390/s18040985

**Published:** 2018-03-26

**Authors:** Tao Zeng, Shaoqiang Chang, Huayu Fan, Quanhua Liu

**Affiliations:** 1 Radar Research Laboratory, School of Information and Electronics, Beijing Institute of Technology, Beijing 100081, China; zengtao@bit.edu.cn (T.Z.); changshaoqiang@bit.edu.cn (S.C.); 2 Department of Electronic Engineering, Tsinghua University, Beijing 100084, China; fan_huayu@sina.com; 3 Key Laboratory of Electronic and Information Technology in Satellite Navigation, (Beijing Institute of Technology), Ministry of Education, Beijing 100081, China

**Keywords:** chaotic signal, stepped frequency, FSK/PSK, low probability of intercept (LPI)

## Abstract

The linear stepped frequency and linear frequency shift keying (FSK) signal has been widely used in radar systems. However, such linear modulation signals suffer from the range–Doppler coupling that degrades radar multi-target resolution. Moreover, the fixed frequency-hopping or frequency-coded sequence can be easily predicted by the interception receiver in the electronic countermeasures (ECM) environments, which limits radar anti-jamming performance. In addition, the single FSK modulation reduces the radar low probability of intercept (LPI) performance, for it cannot achieve a large time–bandwidth product. To solve such problems, we propose a novel chaos-based stepped frequency (CSF) synthesized wideband signal in this paper. The signal introduces chaotic frequency hopping between the coherent stepped frequency pulses, and adopts a chaotic frequency shift keying (CFSK) and phase shift keying (PSK) composited coded modulation in a subpulse, called CSF-CFSK/PSK. Correspondingly, the processing method for the signal has been proposed. According to our theoretical analyses and the simulations, the proposed signal and processing method achieve better multi-target resolution and LPI performance. Furthermore, flexible modulation is able to increase the robustness against identification of the interception receiver and improve the anti-jamming performance of the radar.

## 1. Introduction

Low probability of intercept (LPI) is important for modern radar survival [1,2,3]. The LPI radar typically uses a frequency shift keying (FSK) or phase shift keying (PSK) modulation signal. The PSK signal has the advantages of waveform agility and excellent anti-jamming capability, but suffers from Doppler mismatch problems [4]. The FSK signal can easily achieve a large bandwidth and larger main lobe-to-peak-sidelobe ratio [5]. The most important limitation is that single FSK or PSK modulation step cannot attain a large time–bandwidth product [6,7], thereby limiting the anti-intercept ability of the LPI radar. FSK and PSK composited coded modulation can obtain large time–bandwidth products, achieve complementary advantage between the single-modulation methods, and improve the range resolution and velocity resolution [8,9,10]. FSK/PSK composited coded modulation can significantly improve the LPI performance of radar [11,12].

In addition, many types of radar adopt stepped frequency modulation to increase the signal bandwidth, reduce the system instantaneous bandwidth [13,14] and reduce the challenges associated with modulation technology. For either stepped frequency or frequency-coded applications, linear modulation produces a range–Doppler coupling effect. A previous study [15,16] used Costas sequences to solve this problem. The maximum ambiguity sidelobe is the reciprocal of the coded sequence length. Several methods exist to construct a Costas sequence. Although the Costas array is inherent to any positive integer, these analytical methods are typically limited to prime numbers [17,18]. Also, in the electronic countermeasures (ECM) environments, the Costas frequency-hopping code is relatively fixed, and can be predicted in a straightforward manner using the interceptor to implement precise interference, which reduces the radar anti-jamming performance and robustness against identification of the interception receiver [19,20,21]. Another previous study [22] presented the random stepped frequency signal. Randomized stepped frequency radar is related to random noise radar [22]. The real random noise radar transmits the noise waveform and spectrum with high range resolution [23,24], but faces many challenges in engineering, including reference signal storage, low signal-to-noise ratios (SNRs), and transmitter–receiver isolation [25]. Consequently, much research is focused on the topic of chaos [26,27,28,29,30]. Chaotic nonlinear dynamics can be considered a physical phenomenon that bridges the regular evolution of systems with random evolution [31]. Chaotic systems are relatively easily controlled and sensitive to initial conditions and have noise-like, random-like, and wideband spectrum features [32]. This approach benefits the system by providing a high sensitivity to initial conditions and a sufficient supply of a reproducible non-correlation pseudorandom signal [33].

Based on the analysis above, we present a new chaos-based stepped frequency (CSF) synthesized wideband signal. The signal introduces chaotic frequency hopping between the coherent stepped frequency pulses and chaotic frequency coded in subpulse FSK/PSK composited modulation, which is called CSF-chaotic frequency shift keying (CFSK)/PSK. Through the reasonable design of chaotic parameters, this approach can effectively overcome the range–Doppler coupling caused by linear frequency modulation and provide a “thumbtack”-shaped average ambiguity function. CFSK/PSK composited modulation in a subpulse can overcome the inherent shortcomings of the two single-modulation methods and improve the Doppler resolution. In theory, chaotic frequency hopping is straightforward to generate, and the sequences are infinite and aperiodic. Because they are more flexible than are the Costas and pseudorandom sequences, the CSF-CFSK/PSK signal offers relatively strong anti-jamming performance.

This paper is organized as follows: Section 1 introduces the LPI radar signal features and technology disadvantages of linear stepped frequency or linear frequency-coded signals; Section 2 builds the CSF-CFSK/PSK signal model and then analyzes the optimal signal parameter design; Section 3 first analyzes the signal ambiguity function and then investigates the range and Doppler resolution and LPI performance; Section 4 presents the CSF-CFSK/PSK signal processing method; Section 5 describes the simulations used to validate the effectiveness of the proposed method; and Section 6 presents the conclusion.

## 2. CSF-CFSK/PSK Signal Model and Parameter Design

### 2.1. Signal Model

The model of the CFSK/PSK signal is expressed as follows:

(1) $$s(t)=\sum_{n=0}^{N-1}\sum_{m=0}^{M-1}a_{nm}\mathrm{rect}\left(\frac{t-m\tau_{c}-n\tau_{s}-\frac{\tau_{c}}{2}}{\tau_{c}}\right)\exp\left(j2\pi f_{bn}t\right)$$

**Figure 1:**
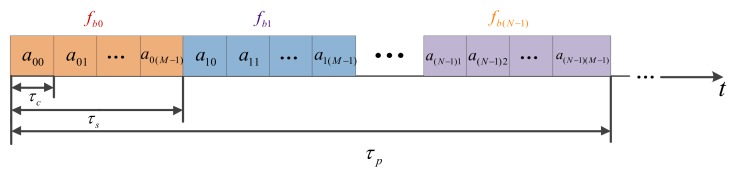
Structure block diagram of chaotic frequency shift keying (CFSK)/phase shift keying (PSK) signal.

The CFSK/PSK uses chaotic frequency-coded modulation in FSK, and each FSK subpulse is modulated by PSK. Figure 1 presents the structure block diagram of CFSK/PSK signal. In the above equation, $\mathrm{rect}\left(t\right)=\left\{ \begin{array}{cc} 1, & \left|t\right|\leq 1/2\\ 0, & \mathrm{Others} \end{array}\right.$, N is the chaotic frequency-coded length of CFSK, M is the phase-coded length of PSK in every CFSK subpulse, $\tau_{c}$ is the phase-coded pulse width, $\tau_{s}=M\tau_{c}$ is the frequency-coded pulse width, $f_{bn}=C_{bn}\Delta f$ are the frequency-coded values, $C_{bn}\in \left\{1,2,3,\cdots ,N\right\}$ is the chaotic sequence, Δf is the stepped frequency in CFSK, and $a_{nm}$ is the $m^{th}$ phase-coded element in the $n^{th}$ frequency-coded subpulse. Various frequency-coded subpulses may have different phase-coded elements.

**Figure 2:**
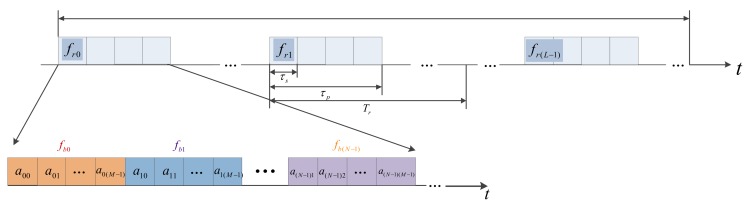
Structure block diagram of chaos-based stepped frequency (CSF)-CFSK/PSK signal.

**Figure 3:**
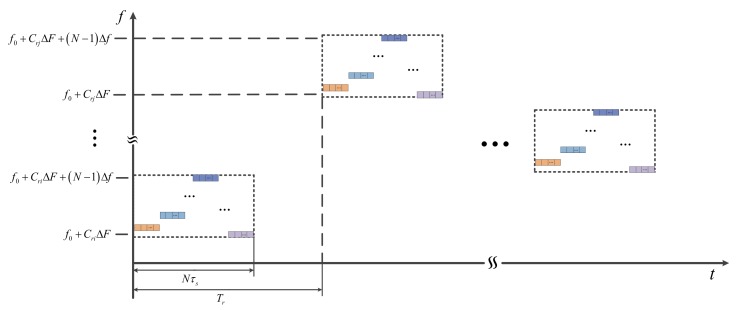
Time–frequency block diagram of CSF-CFSK/PSK signal.

The model of the CSF-CFSK/PSK signal is

(2) $$\begin{array}{l} z\left(t\right)=\;\sum_{l=0}^{L-1}s\left(t-lT_{r}\right)\exp\left(j2\pi f_{rl}t\right)\\ =\sum_{l=0}^{L-1}\sum_{n=0}^{N-1}\sum_{m=0}^{M-1}a_{nm}rect\left(\frac{t-m\tau_{c}-n\tau_{s}-lT_{r}-\frac{\tau_{c}}{2}}{\tau_{c}}\right)\exp\left[j2\pi f_{bn}\left(t-lT_{r}\right)\right]\exp\left(j2\pi f_{rl}t\right) \end{array}$$

where s(t) is the CFSK/PSK subpulse of the stepped frequency; $f_{rl}=f_{0}+C_{rl}\Delta F$ is the carrier frequency of the $l^{th}$ subpulse, $f_{0}$ is the starting stepped frequency, $C_{rl}\in \left\{0,1,2,\cdots ,L-1\right\}$ is the chaotic coded sequence, ΔF is the stepped frequency in CSF, and $T_{r}$ is the pulse repetition time (PRT). The same matching coherent frame has the same subpulse s(t). To enhance the waveform agility and anti-interference ability, various coherent process intervals (CPIs) can follow the disparate parameters, including $a_{nm}$, $f_{bn}$ and $f_{rl}$. The structure block diagram of CSF-CFSK/PSK signal is shown in Figure 2. The time–frequency block diagram is shown in Figure 3.

### 2.2. Parameter Design

#### 2.2.1. FSK/PSK Parameter Design

There are many phase codes in PSK modulation, including binary or polyphase codes. This study uses Baker binary PSK codes in every FSK modulation. The bandwidth of PSK is similar to the single-coded element bandwidth, i.e., $1/\tau_{c}$. The FSK stepped frequency Δf should be smaller than or equal to the PSK bandwidth, i.e., $\Delta f\leq 1/\tau_{c}$. To reduce the high-resolution profile grating lobe of CSF-CFSK/PSK, the stepped frequency ΔF should also be smaller than or equal to the bandwidth of FSK/PSK. In this study, ΔF=NΔf and $\Delta f=1/\tau_{c}$.

#### 2.2.2. Chaotic Frequency-Hopping Sequence Design

**Figure 4:**
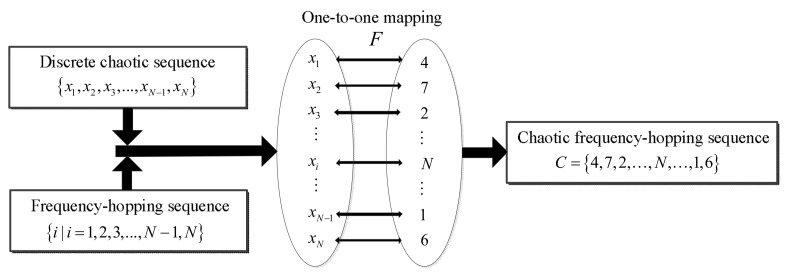
Block diagram of chaotic frequency-hopping sequence generation.

Figure 4 presents the block diagram of chaotic frequency-hopping sequence generation. The challenge lies in the selection of the chaotic mapping and one-to-one mapping F between the discrete chaotic sequence and the frequency-hopping sequence [29]. Different chaotic sequences result in substantial differences in performance in terms of the autocorrelation and the ambiguity function. Therefore, the corresponding chaotic sequences must be selected according to the signal performance requirements. The preferred method of chaotic frequency-hopping sequence generation is to quantify the discrete chaotic mapping trajectory and optimize the quantization threshold, as previously described [34]. These methods must construct the corresponding quantitative criterion according to the various chaotic trajectory characteristics. Because the quantization precision is limited, certain frequency-hopping sequences may fall into the same quantized interval, thereby worsening the coherent processing results. A previous report [35] presented a one-to-one mapping method using queuing theory. In that study, the mapping method was used to generate chaotic frequency-hopping sequences. We use the same method and equate chaotic frequency-hopping sequences with CFSK codes in this study. The mapping method is described as follows:

1. Identify the initial value $x_{1}$ of the chaos mapping and iteration number N and obtain the chaotic sequence $\left\{x_{1},x_{2},\ldots ,x_{N-1},x_{N}\right\}$;
2. Sort the chaotic sequence $\left\{x_{1},x_{2},\ldots ,x_{N-1},x_{N}\right\}$ from small to large to form the new sequence $\left\{x_{l},x_{k},\ldots ,x_{j},\ldots ,x_{p}\right\}$;
3. Find each element decimal position number of the chaotic sequence $\left\{x_{1},x_{2},\ldots ,x_{N-1},x_{N}\right\}$ in the new ordered sequence $\left\{x_{l},x_{k},\ldots ,x_{j},\ldots ,x_{p}\right\}$. Replace the chaotic sequence corresponding element with these decimal location numbers and obtain the decimal number sequence collection $\left\{C_{1},C_{2},\ldots ,C_{N-1},C_{N}\right\}$;
4. The collection $\left\{C_{1},C_{2},\ldots ,C_{N-1},C_{N}\right\}$ is the frequency-hopping sequence obtained using the chaotic sequence $\left\{x_{1},x_{2},\ldots ,x_{N-1},x_{N}\right\}$.

Appendix A lists several common one-dimensional discrete chaotic mapping methods. The modified Bernoulli and modified skew tent sequences are corrected based on the autocorrelation phase-space axial symmetric (APAS) theorem. These two modified sequences have better autocorrelation characteristics than does primal mapping sequences [36].

**Figure 5:**
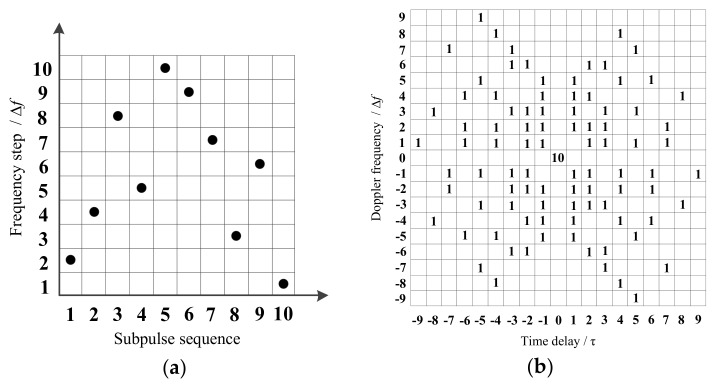
(**a**) Block diagram of the Costas FSK code; (**b**) block diagram of the Costas FSK code ambiguity function.

**Figure 6:**
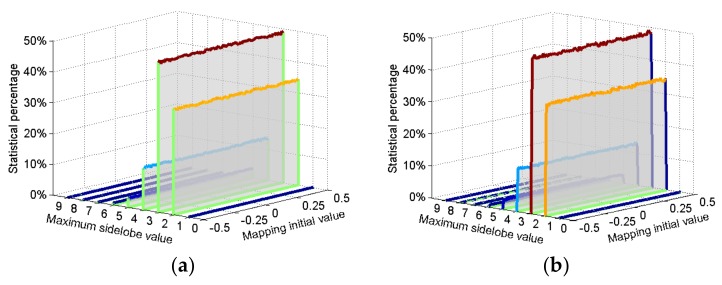
Highest sidelobe statistical percentage of chaotic sequence ambiguity function for various mapping initial value (**a**) modified Bernoulli sequences; (**b**) modified skew tent sequences.

The process is as follows. Allow the chaotic mapping described in Appendix A to iterate 205,000 times for various initial values. Abandon the data from the first 5000 iterations, and then use the remaining 200,000 data values to generate 20,000 groups of frequency-hopping sequences, each with a length of 10 elements. Figure 5 shows the Costas FSK code (length 10) and the block diagram of ambiguity function. Using the same calculation method, count the ambiguity function’s highest sidelobe value for every sequence and the percentage of different highest sidelobe values for 20,000 group chaotic frequency-hopping sequences for the various initial values. The percentage approximates the probability that the maximum sidelobe value appears in ambiguity function. Figure 6 shows the statistical results of the modified Bernoulli and modified skew tent sequences. The variation in initial values of chaotic mapping has a minor effect on the sequence ambiguity function maximum sidelobe level. Appendix B presents the statistical average percentage of the maximum ambiguity function sidelobe level for various chaotic sequences generated by chaotic mapping for various initial values. The modified Bernoulli and modified skew tent mapping has a similar performance to the Gaussian random and uniform random sequences; thus, this study uses these two sequences to obtain the final CFSK codes and frequency-hopping sequences.

**Figure 7:**
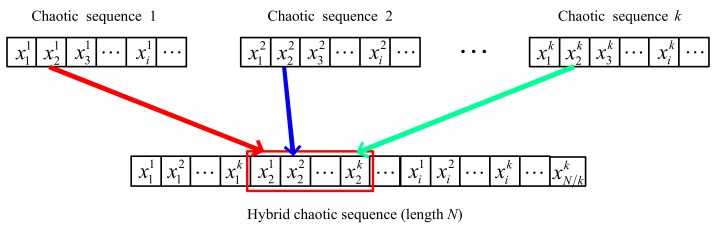
Block diagram of the hybrid chaotic frequency-hopping sequence generation.

**Figure 8:**
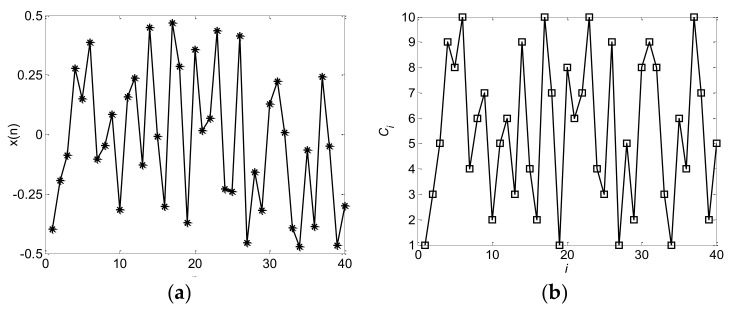
(**a**) Hybrid discrete chaotic mapping trajectory; (**b**) hybrid chaotic frequency-hopping sequences.

Chaotic mapping is sensitive to the initial value, and by changing the initial value, completely different and infinite non-repetitive frequency-hopping sequences can be obtained. By contrast, the chaotic frequency-hopping sequence has the possibility of being predicted. To increase the prediction challenge of the interceptor, two or more discrete chaotic sequences with better performance can be combined to increase the complexity of the sequences, enhance the coding performance [37] and greatly reducing the possibility of prediction. Many methods of obtaining hybrid chaotic sequences are available. In this study, 2 elements from the modified Bernoulli and the modified skew tent sequence are extracted in turn to produce new codes [37]. The block diagram of the hybrid chaotic frequency-hopping sequence generation is shown in Figure 7. The hybrid discrete chaotic mapping trajectory and hybrid chaotic frequency-hopping sequences generated via the modified Bernoulli and modified skew tent are shown in Figure 8. The composited frequency-hopping sequence accurately preserves the characteristics of the hybrid trajectory.

## 3. Ambiguity Function and Resolution Analysis

### 3.1. CSF-CFSK/PSK Ambiguity Function

The CSF-CFSK/PSK signal ambiguity function is

(3) $$\begin{array}{l} \;|\chi_{z}\left(\tau ,\xi \right)|=\left|\int_{-\infty }^{+\infty }z\left(t\right)z^{*}\left(t+\tau \right)e^{j2\pi \xi t}dt\right|\\ =\left|\int_{-\infty }^{+\infty }\left[\sum_{l=0}^{L-1}s\left(t-lT_{r}\right)\exp\left(j2\pi f_{rl}t\right)\right]\left\{\sum_{q=0}^{L-1}s^{*}\left(t+\tau -qT_{r}\right)\exp\left[-j2\pi f_{rq}\left(t+\tau \right)\right]\right\}\exp\left(j2\pi \xi t\right)dt\right|\\ =\left|\sum_{l=0}^{L-1}\sum_{q=0}^{L-1}\int_{-\infty }^{+\infty }s\left(t-lT_{r}\right)s^{*}\left(t+\tau -qT_{r}\right)\exp\left\{j2\pi \left[\left(f_{rl}-f_{rq}\right)t-f_{rq}\tau +\xi t\right]\right\}dt\right|\\ =\left|\sum_{l=0}^{L-1}\sum_{q=0}^{L-1}\begin{array}{l} \exp\left\{j2\pi \left[\left(f_{rl}-f_{rq}\right)lT_{r}-f_{rq}\tau +\xi lT_{r}\right]\right\}\\ \int_{-\infty }^{+\infty }s\left(t\right)s^{*}\left(t+\tau +lT_{r}-qT_{r}\right)\exp\left\{j2\pi \left[\left(f_{rl}-f_{rq}\right)t+\xi t\right]\right\}dt \end{array}\right| \end{array}$$

If $\chi_{b}\left(\tau ,\xi \right)=\int_{-\infty }^{+\infty }s\left(t\right)s^{*}\left(t+\tau \right)\exp\left(j2\pi \xi t\right)dt$ is the CFSK/PSK ambiguity function, then Equation (3) can be derived as Equation (4). The term s(t) represents the CFSK/PSK signal.

(4) $$\begin{array}{l} \;|\chi_{z}\left(\tau ,\xi \right)|\\ =\left|\sum_{l=0}^{L-1}\sum_{q=0}^{L-1}\exp\left\{j2\pi \left[\left(f_{rl}-f_{rq}\right)lT_{r}-f_{rq}\tau +\xi lT_{r}\right]\right\}\chi_{b}\left(\tau +lT_{r}-qT_{r},\xi +f_{rl}-f_{rq}\right)\right|\\ =\left|\begin{array}{l} \sum_{\begin{array}{l} l=0\\ q=l \end{array}}^{L-1}\exp\left[j2\pi \left(\xi lT_{r}-f_{rq}\tau \right)\right]\chi_{b}\left(\tau ,\xi \right)\\ +\sum_{l=0}^{L-1}\sum_{\begin{array}{l} q=0\\ q\neq l \end{array}}^{L-1}\exp\left\{j2\pi \left[\left(f_{rl}-f_{rq}\right)lT_{r}-f_{rq}\tau +\xi lT_{r}\right]\right\}\chi_{b}\left(\tau +lT_{r}-qT_{r},\xi +f_{rl}-f_{rq}\right) \end{array}\right| \end{array}$$

The CFSK/PSK ambiguity function $\left|\chi_{b}\left(\tau ,\xi \right)\right|$ can be derived as

(5) $$\begin{array}{l} \;|\chi_{b}\left(\tau ,\xi \right)|=\left|\int_{-\infty }^{+\infty }s\left(t\right)s^{*}\left(t+\tau \right)e^{j2\pi \xi t}dt\right|\\ =\left|\int_{-\infty }^{+\infty }\begin{array}{l} \left[\sum_{n=0}^{N-1}\sum_{m=0}^{M-1}a_{nm}\mathrm{rect}\left(\frac{t-m\tau_{c}-n\tau_{s}-\frac{\tau_{c}}{2}}{\tau_{c}}\right)\exp\left(j2\pi f_{bn}t\right)\right]\\ \left\{{\sum_{k=0}^{N-1}\sum_{p=0}^{M-1}a_{kp}^{*}\mathrm{rect}\left(\frac{t+\tau -p\tau_{c}-k\tau_{s}-\frac{\tau_{c}}{2}}{\tau_{c}}\right)}^{*}\exp\left[-j2\pi f_{bk}\left(t+\tau \right)\right]\right\}\exp\left(j2\pi \xi t\right)dt \end{array}\right|\\ =\left|\int_{-\infty }^{+\infty }\sum_{n=0}^{N-1}\sum_{m=0}^{M-1}\sum_{k=0}^{N-1}\sum_{p=0}^{M-1}\begin{array}{l} a_{nm}a_{kp}^{*}\mathrm{rect}\left(\frac{t-m\tau_{c}-n\tau_{s}-\frac{\tau_{c}}{2}}{\tau_{c}}\right)\mathrm{rect}{\left(\frac{t+\tau -p\tau_{c}-k\tau_{s}-\frac{\tau_{c}}{2}}{\tau_{c}}\right)}^{*}\\ \exp\left[j2\pi \left(f_{bn}-f_{bk}+\xi \right)t\right]\exp\left(-j2\pi f_{bk}\tau \right)dt \end{array}\right|\\ =\left|\sum_{n=0}^{N-1}\sum_{m=0}^{M-1}\sum_{k=0}^{N-1}\sum_{p=0}^{M-1}a_{nm}a_{kp}^{*}\exp\left(-j2\pi f_{bk}\tau \right)\int_{-\infty }^{+\infty }\begin{array}{l} \mathrm{rect}\left(\frac{t-m\tau_{c}-n\tau_{s}-\frac{\tau_{c}}{2}}{\tau_{c}}\right)\mathrm{rect}{\left(\frac{t+\tau -p\tau_{c}-k\tau_{s}-\frac{\tau_{c}}{2}}{\tau_{c}}\right)}^{*}\\ \exp\left[j2\pi \left(f_{bn}-f_{bk}+\xi \right)t\right]dt \end{array}\right| \end{array}$$

The integral element in Equation (5) can be simplified as

(6) $$\begin{array}{l} \int_{-\infty }^{+\infty }\mathrm{rect}\left(\frac{t-m\tau_{c}-n\tau_{s}-\frac{\tau_{c}}{2}}{\tau_{c}}\right)\mathrm{rect}{\left(\frac{t+\tau -p\tau_{c}-k\tau_{s}-\frac{\tau_{c}}{2}}{\tau_{c}}\right)}^{*}\exp\left[j2\pi \left(f_{bn}-f_{bk}+\xi \right)t\right]dt\\ =\exp\left[j2\pi \left(f_{bn}-f_{bk}+\xi \right)\left(m\tau_{c}+n\tau_{s}\right)\right]\\ \;\int_{-\infty }^{+\infty }\mathrm{rect}\left(\frac{t-\frac{\tau_{c}}{2}}{\tau_{c}}\right)\mathrm{rect}{\left[\frac{t+\tau +\left(m-p\right)\tau_{c}+\left(n-k\right)\tau_{s}-\frac{\tau_{c}}{2}}{\tau_{c}}\right]}^{*}\exp\left[j2\pi \left(f_{bn}-f_{bk}+\xi \right)t\right]dt\\ =\exp\left[j2\pi \left(f_{bn}-f_{bk}+\xi \right)\left(m\tau_{c}+n\tau_{s}\right)\right]\exp\left\{j\pi \left(f_{bn}-f_{bk}+\xi \right)\left[\tau_{c}-\tau -\left(m-p\right)\tau_{c}-\left(n-k\right)\tau_{s}\right]\right\}\\ \;\frac{\sin\left[\pi \left(f_{bn}-f_{bk}+\xi \right)\left(\tau_{c}-\left|\tau +\left(m-p\right)\tau_{c}+\left(n-k\right)\tau_{s}\right|\right)\right]}{\pi \left(f_{bn}-f_{bk}+\xi \right)} \end{array}$$

Note that $\left|\tau +\left(m-p\right)\tau_{c}+\left(n-k\right)\tau_{s}\right|<\tau_{c}$ in Equation (6).

If $F_{d}=f_{bn}-f_{bk}+\xi$, then $t_{d}=\tau +\left(m-p\right)\tau_{c}+\left(n-k\right)\tau_{s}$, and Equation (6) has the similar result as the multicarrier phase coded (MCPC) signal in reference [38]. The CFSK/PSK ambiguity function $\left|\chi_{b}\left(\tau ,\xi \right)\right|$ can be simplified as

(7) $$\begin{array}{l} \;|\chi_{b}\left(\tau ,\xi \right)|\\ =\left|\sum_{n=0}^{N-1}\sum_{m=0}^{M-1}\sum_{k=0}^{N-1}\sum_{p=0}^{M-1}\begin{array}{l} a_{nm}a_{kp}^{*}\exp\left(-j2\pi f_{bk}\tau \right)\exp\left[j2\pi F_{d}\left(m\tau_{c}+n\tau_{s}\right)\right]\\ \exp\left[j\pi F_{d}\left(\tau_{c}-t_{d}\right)\right]\frac{\sin\left[\pi F_{d}\left(\tau_{c}-\left|t_{d}\right|\right)\right]}{\pi F_{d}} \end{array}\right|\\ \;|t_{d}|=\left|\tau +\left(m-p\right)\tau_{c}+\left(n-k\right)\tau_{s}\right|<\tau_{c} \end{array}$$

Equation (7) can be continuously derived as

(8) $$\begin{array}{l} \;|\chi_{b}\left(\tau ,\xi \right)|\\ =\left|\begin{array}{l} \sum_{\begin{array}{l} n=0\\ k=n \end{array}}^{N-1}\sum_{\begin{array}{l} m=0\\ p=m \end{array}}^{M-1}\begin{array}{l} a_{nm}a_{kp}^{*}\exp\left(-j2\pi f_{bk}\tau \right)\exp\left[j2\pi \xi \left(m\tau_{c}+n\tau_{s}\right)\right]\exp\left[j\pi \xi \left(\tau_{c}-\tau \right)\right]\\ \frac{\sin\left[\pi \xi \left(\tau_{c}-\left|\tau \right|\right)\right]}{\pi \xi } \end{array}\\ +\sum_{n=0}^{N-1}\sum_{m=0}^{M-1}\sum_{\begin{array}{l} k=0\\ k\neq n \end{array}}^{N-1}\sum_{\begin{array}{l} p=0\\ p\neq m \end{array}}^{M-1}\begin{array}{l} a_{nm}a_{kp}^{*}\exp\left(-j2\pi f_{bk}\tau \right)\exp\left[j2\pi F_{d}\left(m\tau_{c}+n\tau_{s}\right)\right]\exp\left[j\pi F_{d}\left(\tau_{c}-t_{d}\right)\right]\\ \frac{\sin\left[\pi F_{d}\left(\tau_{c}-\left|t_{d}\right|\right)\right]}{\pi F_{d}} \end{array} \end{array}\right|\\ \;|t_{d}|=\left|\tau +\left(m-p\right)\tau_{c}+\left(n-k\right)\tau_{s}\right|<\tau_{c} \end{array}$$

**Figure 9:**
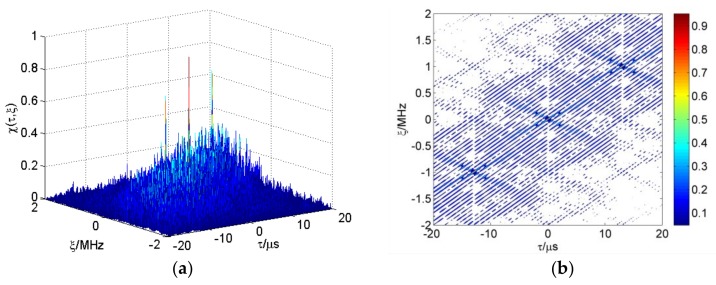
(**a**) Linear frequency shift keying (LFSK)/PSK ambiguity function; (**b**) range–Doppler coupling peak.

**Figure 10:**
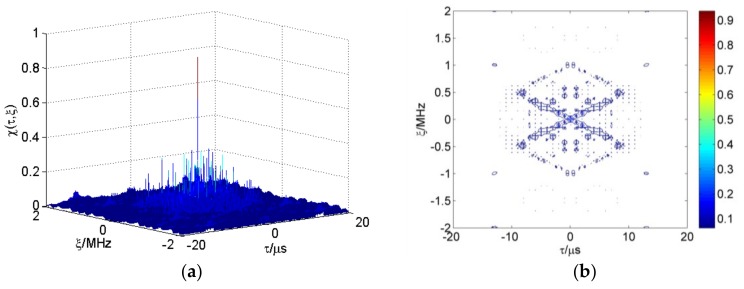
(**a**) CFSK/PSK average ambiguity function; (**b**) thumbtack average ambiguity function.

According to reference [38], the first term of Equation (8) is the autocorrelation element of the CFSK/PSK ambiguity function. The second term is the cross-correlation element. We calculate the average ambiguity function of multiple frame sequences based on Equations (4) and (8). The linear frequency shift keying (LFSK)/PSK ambiguity function is shown in Figure 9a. All FSK adopt 13 Baker binary PSK codes in these figures, with $\tau_{c}=1\;\mu s$. The length N of FSK codes is 10, and Δf=1 MHz. Range–Doppler coupling peaks can be observed on the skew ridge in Figure 9b. The CFSK/PSK average ambiguity function is shown in Figure 10; and this ambiguity function is approximately equal to the ideal thumbtack. Of course, the sidelobe level of CFSK/PSK is still relatively high because of the higher sidelobe level of the Baker binary codes.

**Figure 11:**
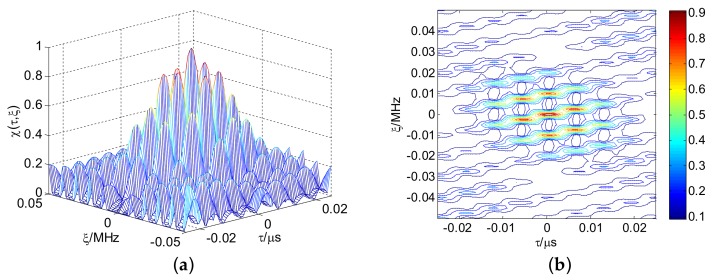
(**a**) LSF-CFSK/PSK average ambiguity function; (**b**) range–Doppler coupling peak.

**Figure 12:**
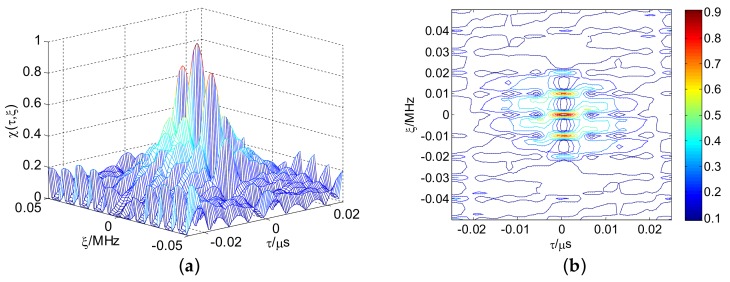
(**a**) CSF-CFSK/PSK average ambiguity function; (**b**) thumbtack average ambiguity function.

Figure 11 presents the linear stepped frequency (LSF)-CFSK/PSK average ambiguity function near the center area. Figure 12 shows the corresponding CSF-CFSK/PSK average ambiguity function. The CFSK/PSK parameters are Baker binary codes ($\tau_{c}=0.25\;\mu s$ and M=13), N=10, and Δf=4 MHz. Moreover, ΔF=40 MHz, $T_{r}=100\;\mu s$, and L=4. Similar to the LFSK/PSK, LSF-CFSK/PSK also has range–Doppler coupling peaks. However, this phenomenon does not occur in the CSF-CFSK/PSK. Moreover, the CSF-CFSK/PSK ambiguity function appears as a thumbtack.

**Figure 13:**
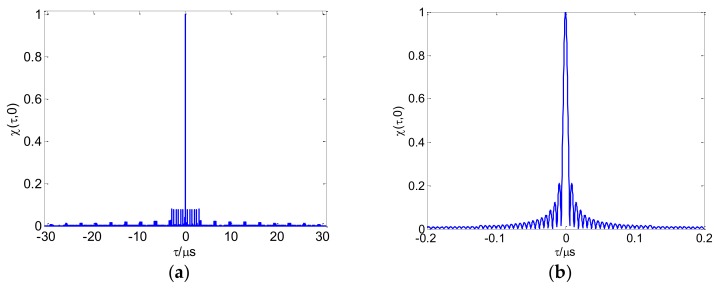
(**a**) CSF-CFSK/PSK range average ambiguity function; (**b**) range average ambiguity function partial enlarged view.

**Figure 14:**
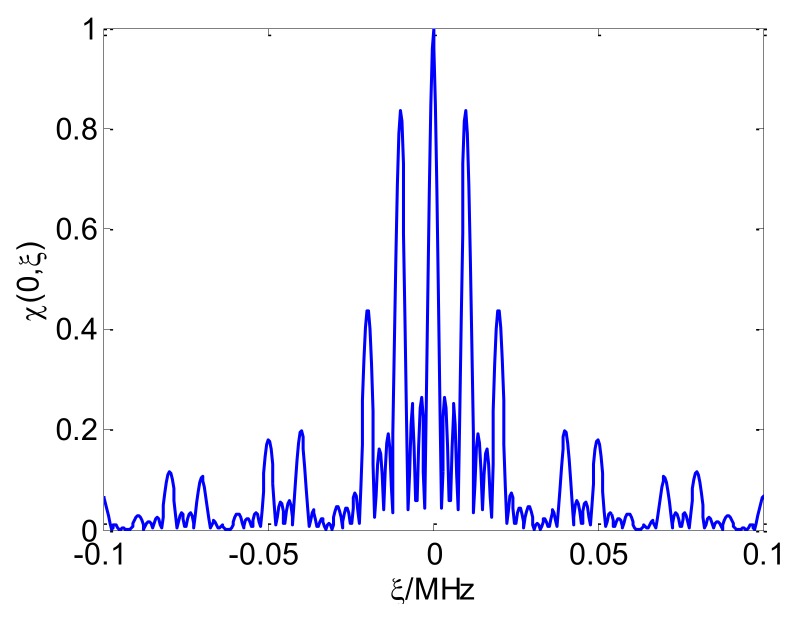
CSF-CFSK/PSK Doppler average ambiguity function.

Figure 13 presents the CSF-CFSK/PSK range average ambiguity function. The range ambiguity function is similar to Sinc in the center, and also contains the grating lobes. The Doppler average ambiguity function in Figure 14 presents a periodic grating in addition to the main lobe. Section 3.2.2 will provide the related analysis.

### 3.2. CSF-CFSK/PSK Resolution Analysis

#### 3.2.1. CFSK/PSK Resolution

Since the ambiguity function of CFSK/PSK is approximately equal to the ideal thumbtack and its main energy is concentrated on the center area. It can be considered that $\left|\tau \right|<\tau_{c}$ and |ξ|<Δf. The second element in Equation (8) is approximated as zero, and the CFSK/PSK range ambiguity function can be simplified as

(9) $$\begin{array}{l} \left|\chi_{b}\left(\tau ,0\right)\right|\approx \left|\tau_{c}-\left|\tau \right|\right|\left|\sum_{\begin{array}{l} n=0\\ k=n \end{array}}^{N-1}\sum_{\begin{array}{l} m=0\\ p=m \end{array}}^{M-1}a_{nm}a_{kp}^{*}\exp\left(-j2\pi f_{bk}\tau \right)\right|=\left|\tau_{c}-\left|\tau \right|\right|\left|\sum_{n=0}^{N-1}\exp\left(-j2\pi f_{bn}\tau \right)\sum_{m=0}^{M-1}a_{nm}a_{nm}^{*}\right|\\ =M\left|\tau_{c}-\left|\tau \right|\right|\left|\sum_{n=0}^{N-1}\exp\left(-j2\pi C_{bn}\Delta f\tau \right)\right|=M\left|\tau_{c}-\left|\tau \right|\right|\left|\frac{\sin\left(\pi N\Delta f\tau \right)}{\sin\left(\pi \Delta f\tau \right)}\right| \end{array}$$

We obtain $\left|t_{d}\right|=\left|\tau +\left(m-p\right)\tau_{c}+\left(n-k\right)\tau_{s}\right|=\left|\tau \right|<\tau_{c}$.

Because |τ| is small in Equation (9), $\left|\tau_{c}-\left|\tau \right|\right|\approx \left|\tau_{c}\right|$. When $\tau =\pm \frac{1}{2N\Delta f}$, $\left|\chi_{b}\left(\tau ,0\right)\right|$ is approximately −4 dB relative to $\left|\chi_{b}\left(0,0\right)\right|$. Thus, the CFSK/PSK signal range resolution is $\Delta R=\frac{c}{2N\Delta f}$, where c is the speed of light. Similarly, the Doppler ambiguity function is

(10) $$\begin{array}{l} \;|\chi_{b}\left(0,\xi \right)|\\ =\left|\sum_{\begin{array}{l} n=0\\ k=n \end{array}}^{N-1}\sum_{\begin{array}{l} m=0\\ p=m \end{array}}^{M-1}a_{nm}a_{kp}^{*}\exp\left[j2\pi \xi \left(m\tau_{c}+n\tau_{s}\right)\right]\exp\left(j\pi \xi \tau_{c}\right)\frac{\sin\left(\pi \xi \tau_{c}\right)}{\pi \xi }\right|\\ =\left|\frac{\sin\left(\pi \xi \tau_{c}\right)}{\pi \xi }\right|\left|\sum_{n=0}^{N-1}\sum_{m=0}^{M-1}a_{nm}a_{nm}^{*}\exp\left[j2\pi \xi \left(m+nM\right)\tau_{c}\right]\right|\\ =\left|\frac{\sin\left(\pi \xi \tau_{c}\right)}{\pi \xi }\right|\left|\sum_{i=0}^{N•M-1}\exp\left(j2\pi \xi i\tau_{c}\right)\right|=\left|\frac{\sin\left(\pi \xi \tau_{c}\right)}{\pi \xi }\right|\left|\frac{\sin\left(\pi \xi \tau_{c}NM\right)}{\sin\left(\pi \xi \tau_{c}\right)}\right| \end{array}$$

We obtain $\left|t_{d}\right|=\left|\tau +\left(m-p\right)\tau_{c}+\left(n-k\right)\tau_{s}\right|=\left|\tau \right|<\tau_{c}$.

Because |ξ| is small, $\left|\frac{\sin\left(\pi \xi \tau_{c}\right)}{\pi \xi }\right|\approx \tau_{c}$ in Equation (10). When $\xi =\pm \frac{1}{2NM\tau_{c}}$, $\left|\chi_{b}\left(0,\xi \right)\right|$ is approximately −4 dB relative to $\left|\chi_{b}\left(0,0\right)\right|$. Thus, the CFSK/PSK signal Doppler resolution is $\Delta \xi =\frac{1}{NM\tau_{c}}$. The velocity resolution is $\Delta v=\frac{\lambda }{2NM\tau_{c}}$, where λ is the transmitting carrier frequency wavelength.

#### 3.2.2. CSF-CFSK/PSK Resolution

The ambiguity function of CSF-CFSK/PSK is also approximately equal to the ideal thumbtack shape. In practical applications, the resolution performance with a small time delay and frequency shift is of more concern. Under this condition, $\left|\tau \right|<T_{r}$, |ξ|<ΔF and q=l in Equation (4). The ambiguity function $\left|\chi_{b}\left(\tau ,\xi \right)\right|$ of CFSK/PSK is approximately equal to the ideal thumbtack shape; thus, $\chi_{b}\left(\tau +lT_{r}-qT_{r},\xi +f_{rl}-f_{rq}\right)\approx 0,q\neq l$ in Equation (4). As a result, the CSF-CFSK/PSK ambiguity function in the central area can be simplified as

(11) $$\left|\chi_{z}\left(\tau ,\xi \right)\right|\approx \left|\sum_{l=0}^{L-1}\exp\left[j2\pi \left(\xi lT_{r}-f_{rl}\tau \right)\right]\chi_{b}\left(\tau ,\xi \right)\right|=\left|\chi_{b}\left(\tau ,\xi \right)\right|\left|\sum_{l=0}^{L-1}\exp\left[j2\pi \left(\xi lT_{r}-f_{rl}\tau \right)\right]\right|$$

The central area ambiguity function envelope of the CSF-CFSK/PSK signal depends mainly on the CFSK/PSK signal, which can be obtained from Equation (11). The CSF-CFSK/PSK range ambiguity function is

(12) $$\begin{array}{l} \left|\chi_{z}\left(\tau ,0\right)\right|\approx \left|\chi_{b}\left(\tau ,0\right)\right|\left|\sum_{l=0}^{L-1}\exp\left(-j2\pi f_{rl}\tau \right)\right|=\left|\chi_{b}\left(\tau ,0\right)\right|\left|\sum_{l=0}^{L-1}\exp\left(-j2\pi l\Delta F\tau \right)\right|\\ =\left|\chi_{b}\left(\tau ,0\right)\right|\left|\frac{\sin\left(\pi L\Delta F\tau \right)}{\sin\left(\pi \Delta F\tau \right)}\right| \end{array}$$

When |τ| is small, $\left|\chi_{b}\left(\tau ,0\right)\right|\approx \left|\chi_{b}\left(0,0\right)\right|$. Similar to the CFSK/PSK signal, $\left|\chi_{z}\left(\tau ,0\right)\right|$ is approximately −4 dB when $\tau =\pm \frac{1}{2L\Delta F}$ relative to $\left|\chi_{z}\left(0,0\right)\right|$. The CSF-CFSK/PSK signal range resolution is $\Delta R=\frac{c}{2L\Delta F}$. The CSF-CFSK/PSK Doppler ambiguity function is

(13) $$\left|\chi_{z}\left(0,\xi \right)\right|\approx \left|\chi_{b}\left(0,\xi \right)\right|\left|\sum_{l=0}^{L-1}\exp\left(j2\pi \xi lT_{r}\right)\right|=\left|\chi_{b}\left(0,\xi \right)\right|\left|\frac{\sin\left(\pi LT_{r}\xi \right)}{\sin\left(\pi T_{r}\xi \right)}\right|$$

According to the analyses above, the Doppler resolution is $\Delta \xi =\frac{1}{LT_{r}}$. The velocity resolution is $\Delta v=\frac{\lambda }{2LT_{r}}$. Note that $\left|\chi_{z}\left(0,\xi \right)\right|$ has grating lobes with periodic $1/T_{r}$ because of the fixed PRT, which indicates that the signal still contains velocity measurement blurs.

### 3.3. LPI Performance Analysis

The LPI performance of the radar can be measured using the interception factor α proposed by Schleher [39]. If α<1, then the radar cannot be detected by the interceptor and possesses satisfactory stealth performance. A smaller value of α corresponds to a better LPI performance of the radar. According to intercept factor theory, the interception factor $\alpha \propto \sqrt{1/B\tau }$ [39], where B is the signal bandwidth, and τ is the signal time width. The CSF-CFSK/PSK has the relationship $\Delta f=1/\tau_{c}$; thus, $\alpha \propto \sqrt{1/\Delta fM\tau_{c}}=\sqrt{1/M}$ for single PSK modulation. In addition, $\alpha \propto \sqrt{1/N\Delta fN\tau_{c}}=1/N$ for single CFSK modulation; $\alpha \propto \sqrt{1/N\Delta fNM\tau_{c}}=1/\sqrt{N^{2}M}$ for CFSK/PSK modulation; $\alpha \propto \sqrt{1/LN\Delta fNM\tau_{c}}=1/\sqrt{LN^{2}M}$ for the CSF-CFSK/PSK signal. The above analysis clearly shows that the interception factor α of the CFSK/PSK signal is decreased by a factor of $\sqrt{1/M}$ and 1/N relative to the single CFSK or PSK modulation, respectively. By adopting the stepped frequency synthesized bandwidth accumulation, the interception factor is further reduced by $\sqrt{1/L}$, which improves the LPI performance.

## 4. CSF-CFSK/PSK Signal Processing Method

### 4.1. Echo Model

According to Equation (2), the transmit signal is

(14) $$z\left(t\right)=\sum_{l=0}^{L-1}s\left(t-lT_{r}\right)\exp\left(j2\pi f_{rl}t\right)=\sum_{l=0}^{L-1}s\left(t-lT_{r}\right)\exp\left[j2\pi \left(f_{0}+C_{rl}\Delta F\right)t\right]$$

The echo signal is

(15) $$z_{e}\left(t\right)=\sum_{l=0}^{L-1}s\left[t-lT_{r}-\tau \left(t\right)\right]\exp\left\{j2\pi \left(f_{0}+C_{rl}\Delta F\right)\left[t-\tau \left(t\right)\right]\right\}$$

The local oscillator signal is

(16) $$z_{r}\left(t\right)=\sum_{l=0}^{L-1}\mathrm{rect}\left(\frac{t-lT_{r}-\frac{T_{r}}{2}}{T_{r}}\right)\exp\left[j2\pi \left(f_{0}+C_{rl}\Delta F\right)t\right]$$

The baseband signal after mixing is

(17) $$\begin{array}{l} z_{B}\left(t\right)=\sum_{l=0}^{L-1}s\left[t-lT_{r}-\tau \left(t\right)\right]\exp\left[-j2\pi \left(f_{0}+C_{rl}\Delta F\right)\tau \left(t\right)\right]\\ =\sum_{l=0}^{L-1}s\left[\left(1+\frac{2v}{c}\right)t-lT_{r}-\tau_{0}\right]\exp\left[-j2\pi \left(f_{0}+C_{rl}\Delta F\right)\tau_{0}\right]\exp\left[j2\pi \left(f_{0}+C_{rl}\Delta F\right)\frac{2v}{c}t\right] \end{array}$$

where τ(t)=2(R−vt)/c, $\tau_{0}=2R/c$, R is the target echo time delay, c is the speed of light, and v is the velocity of the target. The velocity is positive when the target is close to the radar, whereas the velocity is negative when the target is far from the radar. If $t^{\prime }=t-lT_{r}$, then the baseband echo of the l+1 subpulse can be derived according to Equation (18) in the fast time domain.

(18) $$\begin{array}{l} z_{sl}\left(t^{\prime }\right)=s\left[\left(1+\frac{2v}{c}\right)t^{\prime }-\tau_{0}+\frac{2lvT_{r}}{c}\right]\exp\left[-j2\pi \left(f_{0}+C_{rl}\Delta F\right)\tau_{0}\right]\\ \exp\left[j2\pi \left(f_{0}+C_{rl}\Delta F\right)\frac{2v}{c}t^{\prime }\right]\exp\left[j2\pi \left(f_{0}+C_{rl}\Delta F\right)\frac{2lvT_{r}}{c}\right] \end{array}$$

Equation (18) indicates that the target velocity leads to the time scale compression and range walk $2lvT_{r}/c$ of the subpulses. The time scale compression factor is 1+2v/c, and the target delay time and range walk are also compressed by this factor. However, considering v≪c, the compression factor is 1+2v/c≈1. Thus, only the range walk must be corrected. In addition,$\exp\left[-j2\pi \left(f_{0}+C_{rl}\Delta F\right)\tau_{0}\right]$ is the echo time delay phase term, which is mainly used to obtain the target profile; $\exp\left[j2\pi \left(f_{0}+C_{rl}\Delta F\right)2vt^{\prime }/c\right]$ is the intra-pulse Doppler frequency term of the subpulse, and it causes the range profile of the subpulse to diverge, which must be compensated; and $\exp\left[j2\pi \left(f_{0}+C_{rl}\Delta F\right)2lvT_{r}/c\right]$ is the inter-pulse fixed Doppler phase term. Because the relationship between $C_{rl}$ and l is nonlinear, a divergence of the high-resolution range profile arises in coherent processing, and it must be corrected. This divergence explains why the nonlinear stepped frequency synthesized wideband signal ambiguity function is thumbtack-shaped, i.e., improves the multi-target resolution performance.

### 4.2. Synthesized Wideband Processing Based on Frequency Spectrum Splicing

**Figure 15:**
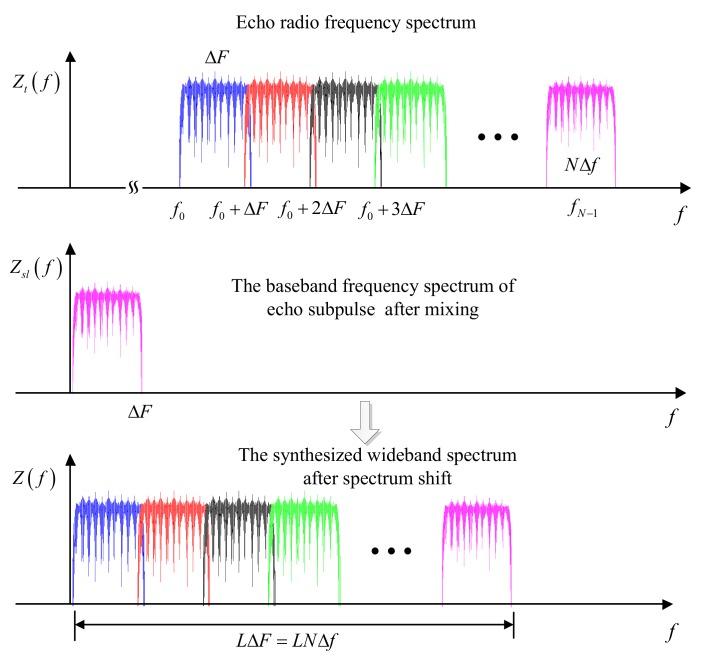
Schematic diagram of spectrum splicing algorithm for the CSF-CFSK/PSK signal.

The spectrum splicing algorithm moves multiple narrowband pulse signals in the frequency domain to synthesize a wideband spectrum and uses weighted inverse matched filtering to achieve a high-resolution range profile [40,41,42]. A schematic diagram of spectrum splicing algorithm for the CSF-CFSK/PSK signal is shown in Figure 15. The algorithm is as follows.

Assuming that the target velocity is 0, the baseband echo of Equation (18) is

(19) $$z_{sl}\left(t^{\prime }\right)=s\left(t^{\prime }-\tau_{0}\right)\exp\left[-j2\pi \left(f_{0}+C_{rl}\Delta F\right)\tau_{0}\right]$$

Taking the Fourier transform of Equation (19) in the fast time domain, the following spectrum is obtained:

(20) $$Z_{sl}\left(f\right)=S\left(f\right)\exp\left(-j2\pi f\tau_{0}\right)\exp\left[-j2\pi \left(f_{0}+C_{rl}\Delta F\right)\tau_{0}\right]$$

where S(f) is the frequency spectrum of $s\left(t^{\prime }\right)$. According to the stepped frequency sequence $C_{rl}$, the $l^{th}$ subpulse spectrum $Z_{sl}\left(f\right)$ is shifted by $C_{rl}\Delta F$ in the frequency domain. After coherent synthesis, the wideband spectrum is

(21) $$\begin{array}{l} Z(f)=\sum_{l=0}^{L-1}Z_{sl}(f-C_{rl}\Delta F)=\exp\left[-j2\pi \left(f+f_{0}\right)\tau_{0}\right]\sum_{l=0}^{L-1}S(f-C_{rl}\Delta F)\\ =\exp\left[-j2\pi \left(f+f_{0}\right)\tau_{0}\right]\sum_{i=0}^{L-1}S(f-i\Delta F) \end{array}$$

When the subpulse bandwidth is B=NΔf=ΔF, the coherent synthesized frequency spectrum is continuous. However, the amplitude spectrum of S(f) contains a ripple at the top and the spectrum beyond the effective bandwidth also affects the flatness of synthesized spectrum, thereby affecting the coherent processing profile. A better solution is to construct the weighted inverse matched filter H(f) to compensate for the amplitude spectrum fluctuation. H(f) can be constructed as [40,42]

(22) $$H(f)=\left\{ \begin{array}{l} {S^{\prime }}^{*}\left(f\right)/\left|S^{\prime }\left(f_{H}\right)\right|,\;f\geq f_{H}\\ {S^{\prime }}^{*}\left(f\right)/{\left|S^{\prime }\left(f\right)\right|}^{2},\;f_{L}\leq f\leq f_{H}\\ {S^{\prime }}^{*}\left(f\right)/\left|S^{\prime }\left(f_{L}\right)\right|,\;f\leq f_{L} \end{array}\right.$$

where $f_{L}$ and $f_{H}$ are the minimum and maximum frequencies in the effective synthesized bandwidth, respectively; and $S^{\prime }\left(f\right)$ is obtained by coherent superposition of the reference subpulse spectrum S(f) and is given by Equation (23). Note that H(f) is no longer a simple complex conjugate of the reference signal spectrum; specifically, H(f) is weighted by ${1/\left|S^{\prime }\left(f\right)\right|}^{2}$ [42].

(23) $$S^{\prime }\left(f\right)=\sum_{i=0}^{L-1}S(f-i\Delta F)$$

Using Equation (22) to filter Equation (21), we obtain

(24) $$\begin{array}{ll} P(f) & =Z(f)H\left(f\right)\\ & =\frac{{S^{\prime }}^{*}\left(f\right)}{{\left|S^{\prime }\left(f\right)\right|}^{2}}\cdot S^{\prime }\left(f\right)\exp\left[-j2\pi \left(f+f_{0}\right)\tau_{0}\right]\\ & =\mathrm{rect}\left(\frac{f-f_{m}^{\prime }}{B_{t}}\right)\exp\left[-j2\pi \left(f+f_{0}\right)\tau_{0}\right],\;f_{L}\leq f\leq f_{H} \end{array}$$

where $B_{t}$ is the effective bandwidth of the synthesized spectrum and $f_{m}^{\prime }$ is the central frequency of $B_{t}$. Performing the inverse Fourier transform, we obtain a high-resolution range profile as given in Equation (25). The peak value of high-resolution profile appears at $\tau_{0}$. The −4 dB main lobe width is $1/B_{t}$.

(25) $$p\left(t\right)=FT^{-1}\left[P\left(f\right)\right]=\mathrm{sinc}\left[B_{t}\left(t-\tau_{0}\right)\right]\exp\left(2\pi f_{m}^{\prime }t\right)\exp\left[-j2\pi \left(f_{0}+f_{m}^{\prime }\right)\tau_{0}\right]$$

If the target velocity is not equal to zero and is independent of the effect of the time scale compression factor, then the synthesized frequency spectrum without compensations and corrections is given by Equation (26).

(26) $$\begin{array}{l} Z(f)=\sum_{l=0}^{L-1}Z_{sl}(f-C_{rl}\Delta F)\\ =\sum_{l=0}^{L-1}S\left(f-f_{dl}-C_{rl}\Delta F\right)\exp\left[-j2\pi \left(f-f_{dl}+f_{0}\right)\tau_{0}\right]\exp\left[j2\pi \left(f-f_{dl}\right)\frac{2lvT_{r}}{c}\right]\exp\left(j2\pi f_{0}\frac{2lvT_{r}}{c}\right) \end{array}$$

$f_{dl}=\left(f_{0}+C_{rl}\Delta F\right)2v/c$ is the Doppler frequency shift produced by the subpulse carrier frequency. Because $f_{dl}$ occurs, the synthesized spectrum is mismatched with the reference spectrum and coupled to the phase term caused by the inter-pulse Doppler and the range walk. The matched filtering result is diverged, and the sidelobe rises when the previous weighted inverse filter is directly used. Thus, compensations and corrections are necessary before the spectrum splicing.

**Figure 16:**
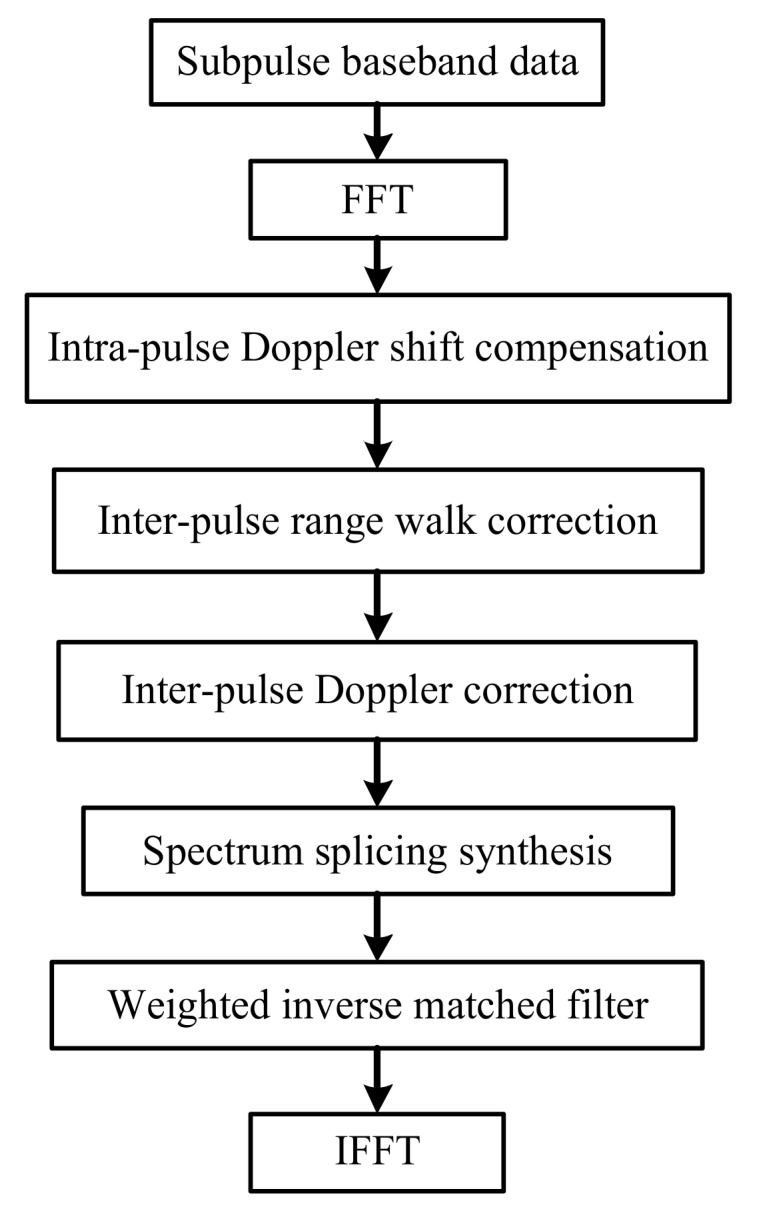
Flow chart of CSF-CFSK/PSK signal synthesized wideband processing method based on the frequency spectrum splicing.

The above derivation is based on the condition of subpulse bandwidth B=NΔf=ΔF. When B=NΔf>ΔF, the spectrum of subpulses are overlapped, and the frequency spectrum must be divided using the splicing processing method. When B=NΔf<ΔF, the synthesized frequency spectrum appears concave or contains gaps, which causes the grating lobe level of the matched result to be prominently raised. NΔf<ΔF should be avoided in the design of parameters. According to the analyses above, the flow chart of CSF-CFSK/PSK signal synthesized wideband processing method based on the frequency spectrum splicing is shown in Figure 16 and described as follows:

1. Obtain the subpulse baseband data and take the fast Fourier transformation (FFT) in time domain;
2. Compensate the intra-pulse Doppler frequency shift $f_{dl}=\left(f_{0}+C_{rl}\Delta F\right)2v/c$;
3. Correct the inter-pulse range walk phase term $\exp\left(j\pi f2lvT_{r}/c\right)$ and the inter-pulse Doppler phase term $\exp\left[j2\pi \left(f_{0}+C_{rl}\Delta F\right)2lvT_{r}/c\right]$;
4. Take the spectrum splicing synthesis and the weighted inverse matched filter processing;
5. Perform the inverse fast Fourier transformation (IFFT) for the synthesized wideband spectrum and get the high-resolution range profile.

## 5. Simulation and Discussion

The processing simulation parameter of CSF-CFSK/PSK signal is shown in Table 1.

**Figure 17:**
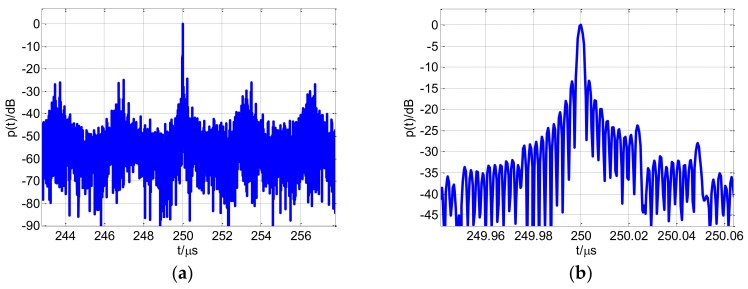
(**a**) Synthesized wideband high-resolution range profile; (**b**) range profile partial enlarged view.

**Figure 18:**
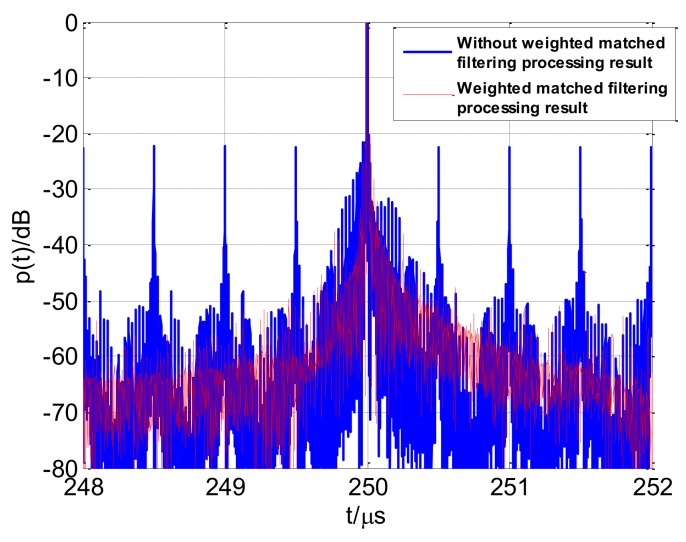
Comparison of various matched filtering imaging results.

The synthesized wideband high-resolution range profile is shown in Figure 17. The profile in Figure 17a has 2N−1 grating lobes with a period of approximately $\tau_{c}M=3.25\;\mu s$, and the amplitude of the grating lobes is lower than that of the main lobe. The period and amplitude of these grating lobes exactly correspond to the CSF-CFSK/PSK range average ambiguity function, but are consistent with Equation (25), which is primarily because the actual target echo frequency spectrum is not the ideal rectangle after performing compensation, corrections, and weighted inverse matched filtering. However, as shown in Figure 18, the weighted inverse matched filtering can significantly reduce the grating lobes level around the main lobe. The period of these grating lobes is $\tau_{c}2=0.5\;\mu s$. Figure 17b shows the main lobe of the high-resolution range profile.

**Figure 19:**
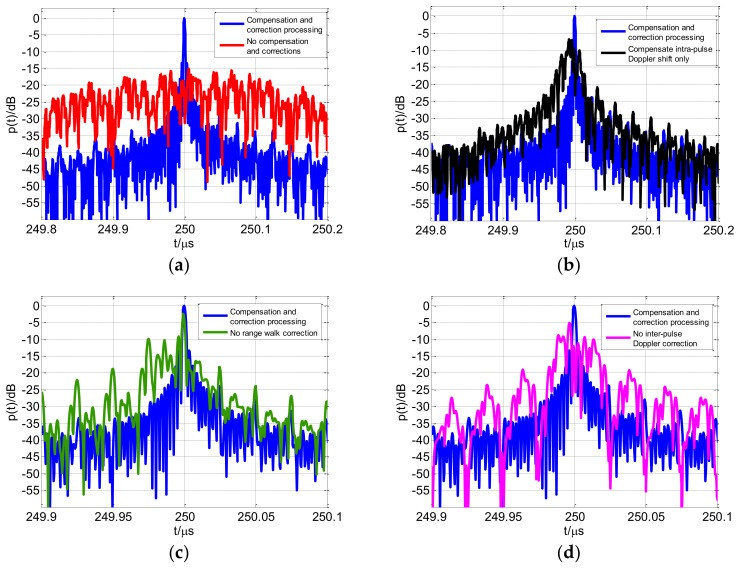
Processing results of various compensation and correction conditions (**a**) no compensation and corrections; (**b**) compensation for intra-pulse Doppler shift only; (**c**) no range walk correction; (**d**) no inter-pulse Doppler correction.

The profiles using the various compensation and correction conditions are shown in Figure 19. If Doppler and range walk are not compensated for and corrected, respectively, then the high-resolution profile diverges. Relative to the other conditions, ignoring the range walk correction has fewer effects on the profile. The profile image has a certain divergence; however, the main lobe can still be identified. Compensating for the intra-pulse Doppler shift only or ignoring the inter-pulse Doppler correction also lead to divergence in the coherent processing range profile. Figure 19 indicates that the flow chart in Figure 16, and the processing methods proposed are effective.

**Figure 20:**
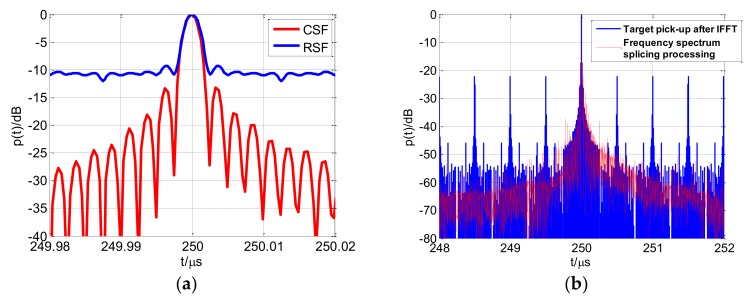
High-resolution range profile comparisons (**a**) random stepped frequency (RSF) and CSF; (**b**) Target pick-up after inverse fast Fourier transformation (IFFT) and frequency spectrum splicing processing.

The previous study [22] analyzed the random stepped frequency (RSF) signal. The received signal vector of RSF is correlated with a reference signal to achieve high-resolution range profile, and the correlation output is accompanied by a random noise component, which increases the sidelobe level [22]. Figure 20a presents the high-resolution range profile of RSF and CSF. The RSF output adopts Monte Carlo simulation with the Gaussian distribution random frequency hopping and the results indicate that CSF possesses better coherent performance. The study [29] proposed the chaotic-based random stepped frequency (CRSF) signal. The CRSF signal also introduced chaotic frequency hopping between the coherent stepped frequency pulses but adopted simple rectangular pulse modulated in subpulses. The received echo vector could be rearranged to the linear order according to the transmitted frequency-hopping sequence in CRSF. Therefore, the target pick-up algorithm based on IFFT was used to obtain the high-resolution range profile. For the CSF-CFSK/PSK signal, frequency spectrum splicing method possesses larger computational burden than the target pick-up algorithm based on IFFT, but can effectively suppress the profile grating lobes around main lobe through the weighted inverse matched filter processing. The high-resolution range profile in Figure 20b demonstrates the superiority of the method we proposed.

## 6. Conclusions

We have proposed a chaos-based stepped frequency synthesized wideband signal. The novel CSF-CFSK/PSK signal introduces chaotic frequency hopping between the coherent stepped frequency pulses and chaotic frequency coded in subpulse FSK/PSK composited modulation. Theoretical analyses and the simulations show that the signal exhibits a “thumbtack”-shaped ambiguity function with the optimized discrete chaotic mapping and chaotic frequency-hopping sequence generation method, thus improving the radar’s multi-target resolution. Moreover, the CSF-CFSK/PSK signal can overcome the inherent shortcomings of single FSK or PSK modulation and attain a better LPI performance. In theory, chaotic frequency hopping can be generated easily, and the sequences are infinite and aperiodic. Thus, the proposed signal is more flexible than are Costas and pseudorandom sequences, and has better anti-jamming performance. In addition, a synthesized wideband coherent processing method based on frequency spectrum splicing was presented for the CSF-CFSK/PSK signal. The simulation results demonstrate the effectiveness and the superiority of the method we proposed.

## Figures and Tables

**Table 1:** Signal processing simulation parameters.

Parameter	Value
$\tau_{c}$	0.25 μs
M	13
N	10
L	10
Δf	4 MHz
ΔF	40 MHz
$f_{0}$	10 GHz
$T_{r}$	500 μs
v	500 m/s
$\tau_{0}$	250 μs

## Appendix A

**Table A1:** Selected one-dimensional discrete chaotic mapping examples (here, ε is the minimal positive number).

**Chaotic Mapping**	**Bernoulli**	**Modified Bernoulli**
Iterative formula	$\begin{array}{l} \left\{ \begin{array}{l} x\left(n+1\right)=bx\left(n\right)+a,x\left(n\right)<0\\ x\left(n+1\right)=bx\left(n\right)-a,x\left(n\right)\geq 0 \end{array}\right.\\ a=0.5;\;b=2-\varepsilon \\ x\left(n\right)\in \left(-0.5,0.5\right) \end{array}$	$\begin{array}{l} \left\{ \begin{array}{l} x\left(n+1\right)=bx\left(n\right)+1.5,\;\mathrm{if}\;-0.5\leq x\left(n\right)<-0.25\\ x\left(n+1\right)=bx\left(n\right)+0.5,\;\mathrm{if}\;-0.25\leq x\left(n\right)<0\\ x\left(n+1\right)=-bx\left(n\right)+0.5,\;\mathrm{if}\;0\leq x\left(n\right)<0.25\\ x\left(n+1\right)=-bx\left(n\right)+1.5,\;\mathrm{if}\;0.25\leq x\left(n\right)<0.5 \end{array}\right.\\ b=4-\varepsilon ;\;x\left(n\right)\in \left[-0.5,0.5\right] \end{array}$
**Chaotic Mapping**	**Logistic**	**Tent**
Iterative formula	$\begin{array}{l} x\left(n+1\right)=b\left[a^{2}-x^{2}\left(n\right)\right]-a\\ a=0.5;\;b=4-\varepsilon \\ x\left(n\right)\in \left(-0.5,0.5\right) \end{array}$	$\begin{array}{l} x\left(n+1\right)=a-b\left|x\left(n\right)\right|\\ a=0.5;\;b=2-\varepsilon \\ x\left(n\right)\in \left(-0.5,0.5\right) \end{array}$
**Chaotic Mapping**	**Skew Tent**	**Modified Skew Tent**
Iterative formula	$\begin{array}{l} \left\{ \begin{array}{l} y\left(n+1\right)=\frac{y\left(n\right)}{a}\;\mathrm{if}\;0\text{<}y\left(n\right)\leq a\\ y\left(n+1\right)=\frac{y\left(n\right)-1}{a-1}\;\mathrm{if}\;a\text{<}y\left(n\right)\leq 1\\ x\left(n+1\right)=y\left(n+1\right)-0.5 \end{array}\right.\\ \;x\left(n\right)\in \left(-0.5,0.5\right);\;a\in \left(0,1\right) \end{array}$	$\begin{array}{l} \left\{ \begin{array}{l} y\left(n+1\right)=\frac{y\left(n\right)}{a},\;\mathrm{if}\;0\leq y\left(n\right)\leq a\\ y\left(n+1\right)=\frac{y\left(n\right)-0.5}{a-0.5},\;\mathrm{if}\;a<y\left(n\right)\leq 0.5\\ y\left(n+1\right)=\frac{y\left(n\right)-0.5}{0.5-a},\;\mathrm{if}\;0.5\text{<}y\left(n\right)\leq 1-a\\ y\left(n+1\right)=-\frac{y\left(n\right)-1}{a},\;\mathrm{if}\;1-a<y\left(n\right)\leq 1\\ x\left(n+1\right)=y\left(n+1\right)-0.5 \end{array}\right.\\ x\left(n\right)\in \left[-0.5,0.5\right];\;a\in \left(0,0.5\right) \end{array}$

## Appendix B

**Table A2:** Statistical average percentage of the maximum ambiguity function sidelobe level for various chaotic sequences generated by chaotic mapping for various initial values.

**Sidelobe Level**	**Bernoulli**	**Logistic**	**Tent**	**Skew Tent**
1	0.48%	0%	0%	0%
2	10.2%	23.35%	21.78%	1.89%
3	33.26%	42.3%	43.17%	10.57%
4	28.94%	19.36%	19.67%	19.88%
5	15.54%	9.38%	9.63%	16.1%
6	7.39%	3.34%	3.43%	14.12%
7	2.85%	1.18%	1.21%	6.09%
8	0.94%	0.83%	0.83%	17.64%
9	0.4%	0.26%	0.26%	13.71%
**Sidelobe Level**	**Modified Bernoulli**	**Modified Skew Tent**	**Gaussian Random**	**Uniform Random**
1	0.055743%	0.07055%	0.057%	0.074%
2	34%	34.04%	39.07%	38.82%
3	48.56%	49.3467%	49.4%	49.66%
4	13.55%	12.97%	10.123%	10.04%
5	3.04%	2.84%	1.23%	1.27%
6	0.64%	0.59%	0.11%	0.128%
7	0.13%	0.11%	0.009%	0.0075%
8	0.0188%	0.0286%	0.001%	0.0005%
9	0.005495%	0.00415%	0%	0%
